# Stepwise approach for the control and eventual elimination of *Taenia solium* as a public health problem

**DOI:** 10.1186/s12879-019-3812-y

**Published:** 2019-02-21

**Authors:** Uffe Christian Braae, Sarah Gabriël, Chiara Trevisan, Lian F. Thomas, Pascal Magnussen, Bernadette Abela-Ridder, Helena Ngowi, Maria Vang Johansen

**Affiliations:** 1 One Health Center for Zoonoses and Tropical Veterinary Medicine, Ross University School of Veterinary Medicine, P.O. Box 334, Basseterre, Saint Kitts and Nevis; 2 Department of Infectious Disease Epidemiology and Prevention, Statens Serum Institut, DK-2300 Copenhagen, Denmark; 3 Department of Veterinary Public Health and Food Safety, Faculty of Veterinary Medicine, Ghent University, 9820 Merelbeke, Belgium; 4 Department of Biomedical Sciences, Institute of Tropical Medicine, Antwerp, Belgium; 5 Institute for Infection & Global Health, University of Liverpool, Neston, UK; 6 International Livestock Research Institute, P.O. Box 30709, Nairobi, Kenya; 7 Department of Veterinary and Animal Sciences, Faculty of Health and Medical Sciences, University of Copenhagen, DK-1870 Frederiksberg, Denmark; 8 Centre for Medical Parasitology, Faculty of Health of Medical Sciences University of Copenhagen, DK-2200 Copenhagen, Denmark; 9 Department of Control of Neglected Tropical Diseases, World Health Organization, 1211 Geneva, Switzerland; 10 Department of Veterinary Medicine and Public Health, Sokoine University of Agriculture, Morogoro, Tanzania

**Keywords:** *Taenia solium*, Taeniosis, Cysticercosis, Control, Neglected tropical disease, Zoonosis, Step-wise approach

## Abstract

**Background:**

*Taenia solium* taeniosis/cysticercosis is a public health and agricultural problem, especially in low-income countries, and has been ranked the top foodborne parasitic hazard globally. In 2012, the World Health Organization published a roadmap that called for a validated strategy for *T. solium* control and elimination by 2015. This goal has not been met, and validated evidence of effective control or elimination in endemic countries is still incomplete. Measuring and evaluating success of control programmes remains difficult, as locally acceptable targets have not been defined as part of the 2012 roadmap nor from other sources, and the performance of tools to measure effect are limited.

**Discussion:**

We believe that an international agreement supported by the tripartite World Health Organization, Food and Agriculture Organization of the United Nations, and World Organisation for Animal Health is needed to facilitate endemic countries in publicising SMART (Specific, Measurable, Achievable/attainable, Relevant, Time-bound) country-level control target goals. These goals should be achievable through locally acceptable adoption of options from within a standardised ‘intervention tool-kit’, and progress towards these goals should be monitored using standardised and consistent diagnostics. Several intervention tools are available which can contribute to control of *T. solium*, but the combination of these - the most effective control algorithm - still needs to be identified. In order to mount control efforts and ensure political commitment, stakeholder engagement and funding, we argue that a stepwise approach, as developed for Rabies control, is necessary if control efforts are to be successful and sustainable.

**Conclusions:**

The stepwise approach can provide the framework for the development of realistic control goals of endemic areas, the implementation of intervention algorithms, and the standardised monitoring of the evaluation of the progress towards obtaining the control target goals and eventually elimination.

## Background

*Taenia solium* taeniosis/cysticercosis is a public health and agricultural problem, especially in low-income countries, and has been ranked the top foodborne parasitic hazard globally [12, 23]. The World Health Assembly passed Resolution the WHA66.12 in May 2013, which called on member states to recognise and support the implementation of the 2012 World Health Organization (WHO) roadmap ‘Accelerating work to overcome the global impact of neglected tropical diseases’. The roadmap called for a validated strategy for *T. solium* control and elimination by 2015 and interventions to be scaled up by 2020 in selected countries [25]. The first of these goals was not met [26], and there is currently no consensus about the most appropriate or effective approach for control, or how to evaluate their success [20]. Control of an infectious disease has been defined as: “a reduction in disease incidence, prevalence, morbidity, or mortality to a locally acceptable level, as a result of deliberate efforts” [11]. Locally acceptable levels have not been defined as part of the WHO 2012 roadmap and therefore measuring and evaluating success of control remains difficult.

## Discussion

### Current control and modelling attempts

We believe that in order to reduce the burden of *T. solium,* specifically morbidity and mortality due to neurocysticercosis (NCC), countries should enter into an agreement facilitated through the tripartite alliance to control this parasite, in a similar manner to those existing on other One Health priority areas. This agreement should include guidelines on how to establish the locally acceptable level of disease occurrence (country level control goals), a description of a standardised ‘intervention tool-kit*’ and guidelines on standardised diagnostics as further discussed below.

Several interventions assessing various options for control of *T. solium* transmission have been trialled [13, 22]. Most of the studies so far have measured control at a single timepoint post intervention without the use of a control/comparison group [7], and been short-lived making the benefits of long-term intervention impossible to quantify. Recently, a large-scale study with the aim of eliminating transmission using a combination of human and pig mass drug administration treatment was carried out in Peru [14], this study indicated that a One Health approach to the control of *T. solium* may be possible, given appropriate resources. Modelling studies also point towards using a One Health approach targeting both porcine and human hosts, as being more likely to succeed compared to a single host intervention strategy [3, 17, 27]. These models also emphasise the need for continuous monitoring over time and indicate to us that an intervention algorithm (combination of interventions from the tool-kit of intervention tools) is needed. With a standardised tool-kit of the existing intervention tools, control is theoretically possible [3], and thus the initiation of a control programme is the next logical step [13].

### Country-level control goals

If the internationally agreed aim is to reduce the incidence (and therefore burden) of NCC, then this should be reflected in publicised country-specific SMART (Specific, Measurable, Achievable/attainable, Relevant, Time-bound) goals. These goals should be achievable through locally acceptable adoption of options from within the standardised ‘intervention tool-kit’, and progress towards these goals should be measured using standardised and consistent diagnostics as outlined in the suggested international tripartite agreement. It is our belief that country-specific goals and control initiatives are needed due to the different biological, sociocultural, and socioeconomic factors, which influence local parasite epidemiology, available resources, and government public health priorities. Differences also exist between countries in health care costs, pork prices, and general financial standards, which result in differing levels of cost-benefit from control programmes. Control initiatives should be horizontal cross-sectoral programmes including at least both public health and livestock/agricultural sectors, and they should run under CONSORT guidelines [21]. It is of vital importance that consensus is achieved on how control efforts are to be monitored and the success of programmes evaluated.

### Standardised monitoring of control programmes

Although locally specific goals will centre on the reduction of NCC incidence, direct monitoring of NCC is difficult due to the lack of sensitive and specific affordable diagnostic tests and the late on-set or absence of symptoms altogether. Reduction in NCC incidence is, therefore only suitable as a long-term indicator of control effect. Short-term monitoring of control must rely on more tangible and rapid measurements such as a reduction in incidence of taeniosis or porcine cysticercosis. Since *T. solium* taeniosis is the sole cause of NCC, the incidence of taeniosis can in theory, be used as a proxy indicator for the risk of NCC, and likewise, can the incidence of porcine cysticercosis, the cause of *T. solium* taeniosis, be used as a proxy indicator for the risk of acquiring taeniosis. Currently there is no model for estimating the proportional relationships between the incidence/prevalence of porcine cysticercosis, taeniosis incidence, and NCC incidence or the influence of a variety of local transmission factors such as the pig production system, latrine provision, or culinary habits. In order, therefore, to use porcine cysticercosis incidence as a true proxy for the incidence of NCC, data describing the relationships between changes in disease incidence of taeniosis and porcine cysticercosis on the impact of NCC incidence is urgently needed. Until data emerges to suggest otherwise, we believe that porcine cysticercosis remains the best short-term indication of changes in active transmission of *T. solium*, as the lifespan of pigs within production systems is short and varies relatively little across regions. The high turnover of pigs means that estimating prevalence of porcine cysticercosis is almost as sufficient as incidence estimation. For both porcine cysticercosis and taeniosis there is a need for consensus on the levels of prevalence/incidence defining high, moderate, and low transmission areas as well as cut off points at which the diseases can be classified as ‘of low public health importance’.

Highly endemic areas of porcine cysticercosis can be determined by tongue examination, which is rapid and of low cost, though has low sensitivity [10]. Carcass dissection is not as a standalone method applicable for monitoring control initiatives as the removal of large numbers of pigs, needed to yield accurate prevalence or incidence estimations, could inadvertently affect transmission dynamics, as well as local pork value chains, prohibiting a long-term measurement of intervention effects. Serological detection tools can be used ante mortem, but the performance is still suboptimal [8, 16], and availability remains an issue in most endemic countries and even more so in rural areas. Development and commercialisation of an affordable, rapid, easy to apply ante mortem diagnostic test with high sensitivity and specificity suitable for use in rural areas is needed [9].

We do not believe, however, that the current paucity of diagnostics should delay the implementation of control initiatives. Test performance must though be taken into consideration when goals of success are set. In order to standardise monitoring of control programmes using existing tools we therefore suggest dividing the monitoring of control effect into three phases – 1) Pre-programme (unknown endemicity), 2) Early-programme (high to moderate endemicity), and 3) Late-programme phase (low endemicity). Each phase will require a specific monitoring approach correlated to the level of endemicity, and will thus change as the level of endemicity changes throughout the programme phases [1].

- Phase one – Identification of the need for control: In this phase, we identify areas of high risk of transmission from pigs to humans. We assume that heavily infected pigs carrying thousands of cysts pose a greater risk than pigs with low intensity infections and we therefore suggest using tongue examination to identify such areas. Due to the ease of implementation and low cost, large areas can be screened at relative low cost. Within communities, pigs more than 6 months of age should be included in the screening.
- Phase two – High to moderate endemicity: Prior to starting a control intervention and at pre-determined points within the intervention the prevalence of moderate and heavily infected pigs should be determined and an estimate made of the infection intensity. Pigs of minimum slaughter weight/age within the community should be screened using serology and a sub-set of positive animals would then undergo full carcass detection [20].
- Phase three – Low endemicity: Once a pre-determined prevalence has been reached, estimates of the prevalence of infection should be made at points of slaughter. Pigs presented for slaughter must undergo an expanded post-mortem examination as described by Lightowlers et al. [19] before being cleared for human consumption. NCC incidence should also be monitored onwards from this point to evaluate long-term benefits of the programme.

### Implementation of a control programme – A stepwise approach

**Fig. 1:**
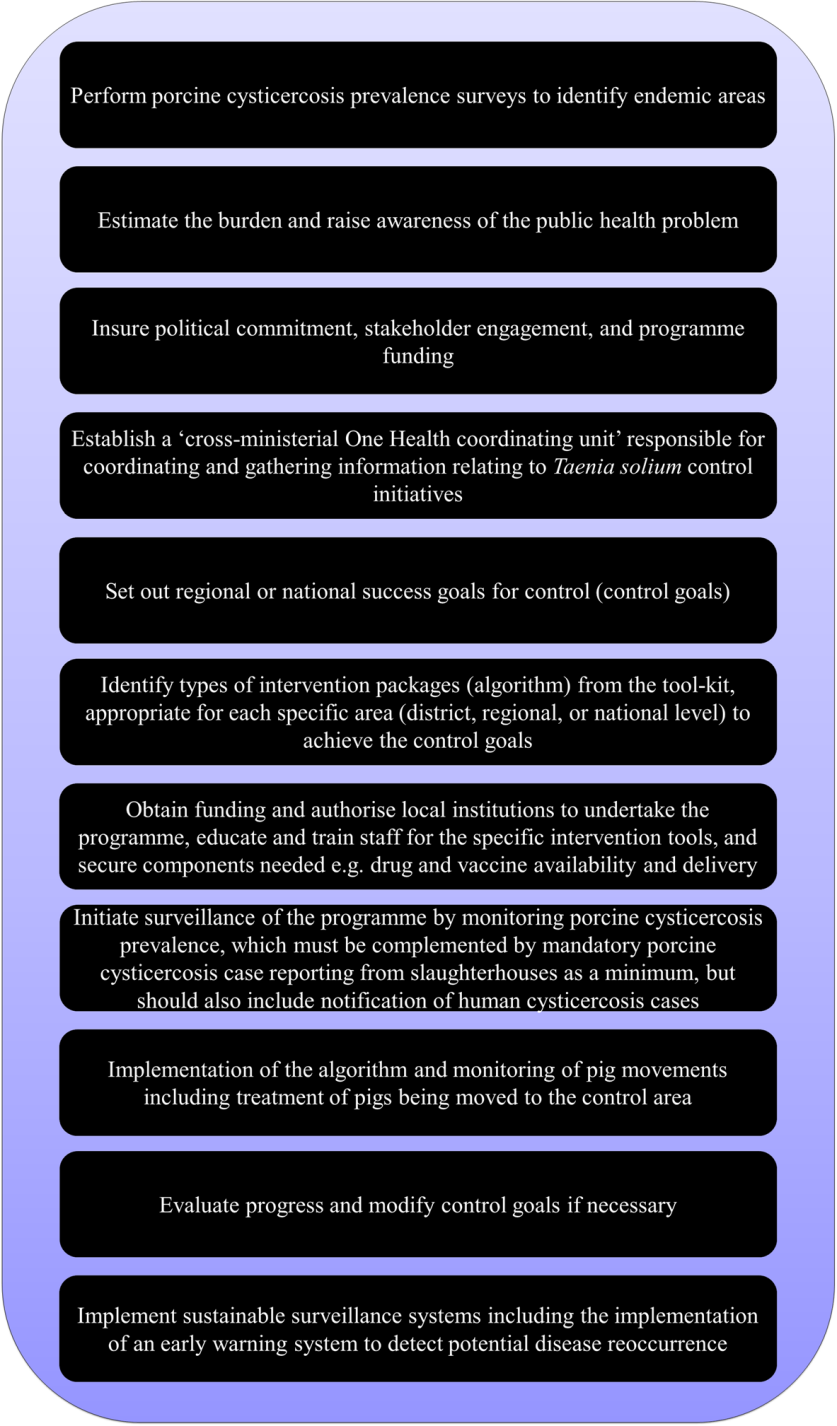
Stepwise approach to sustainable *Taenia solium* control

We suggest that a stepwise approach, as developed for Rabies control [18], is necessary if control efforts are to be successful and sustainable. A step-wise approach is a series of “*measurable steps, designed as a logical flow of activities*” (https://rabiesalliance.org/capacity-building/blueprint-sare) and could be applied to *T. solium* control as illustrated in Fig. 1. A feasible approach is to initiate a programme as a research project, but with cross-sectoral collaboration with relevant stakeholders and authorities on local, regional, and national level, supported by international donors. However, programmes must eventually be administrated and (co-)funded locally to remain sustainable, and cost-effectiveness analyses are essential to justify their adoption and continuation. The role of the private investment (e.g. from pig farmers, pork processors etc.), should also be further investigated as where private goods (such as increased weight gain after the use of anthelmintic treatment, higher prices for a better quality product etc.) are being delivered, certain control strategies may be cost-effective from this perspective.

Initial disease distribution and burden needs to be estimated and the presence and awareness of the parasite recognised (step 1 & 2). This information should lead to securing political commitment, stakeholder engagement, both necessary for programme funding (step 3). Political commitment should facilitate the establishment of a ‘cross-ministerial One Health coordinating unit’ responsible for coordinating and gathering information relating to *T. solium* control initiatives (step 4). This unit should in collaboration with relevant government institutions develop regional or national success goals for control, targets for success and identify timelines for these, based on the international tripartite agreement (step 5). The unit will also identify the country specific control algorithm from the internationally agreed ‘intervention tool kit’, authorise local institutions to undertake the programme, build capacity by training staff, ensure availability of intervention tools such as delivery of drugs and vaccines, and support local institutions to strengthen involvement of the local communities (step 6 & 7). Key intervention tools such as oxfendazole for treatment and a vaccine for prevention of porcine cysticercosis are available for research purposes, but still need registration for use in pigs in many countries where *T. solium* is endemic. Affordability may become an important constraint to the use of a vaccine and a strategy for subsidising the vaccine should be developed. Increasing public awareness through large-scale dissemination of information, and improving education curriculum to professionals should be part of any long-term strategy. The targeted public should include pig keeping households, professionals in the pig sector, schools, and potential consumers including local consumer protection associations if present. Hereafter, surveillance, considered and implemented as an intervention approach [2], of the programme should be initiated by activating standardised porcine cysticercosis prevalence monitoring appropriate to the intervention phase, which must be complemented by mandatory porcine cysticercosis case reporting as a minimum, but should also include notification of human cysticercosis cases (step 8). Local institutions can now implement the algorithm, in conjunction with government support to enforce monitoring of pig movement into control areas (step 9). Monitoring pig movement might also reduce risk of introducing other porcine diseases such as African swine fever into control areas, thus, strengthening the control programme and community compliance. Measures should be taken to implement mandatory treatment of pigs being imported into the control area to avoid reintroduction of cysticercosis. Restricting human movement is impossible and therefore the reintroduction of *T. solium* through taeniosis cases from other endemic areas will remain an issue. Lastly, progress must be evaluated and control goals modified if necessary, including the setting of more ambitious goals for sustained control if applicable (step 10 & 11). Once locally defined goals have been successfully reached, sustainable surveillance systems including the implementation of an early warning system to detect potential disease reoccurrence is needed.

### Tanzania as a pilot country for a stepwise approach

Tanzania is one of the most studied countries in terms of the epidemiology of *T. solium* in sub-Saharan Africa [4, 6, 24], and therefore an obvious country candidate for control programme implementation. It furthermore yields the opportunity for integration with the Tanzanian National Schistosomiasis Control Programme, suggested to have an effect on taeniosis prevalence [5]. In the beginning of 2018, the Tanzanian Government launched the One Health Coordination Desk and the National One Health Strategic Plan, to address emerging health challenges at the animal-environment-human interface. This One Health coordination unit would be an excellent facilitator in the implementation of the ‘stepwise approach’.

## Conclusions

Measuring and evaluating the success of control programmes remains a major issue. Consensus is needed to set forth realistic control goals for endemic areas, and standardise monitoring to evaluate the progress towards those goals. We suggest adhering to the stepwise approach put forward here as a guide towards control and elimination of *T. solium*. While significant progress in addressing *T. solium* control issues has been achieved in the last 10 years, there is still further progress to be made [15], and it is critical that this momentum is kept with continuous tripartite (FAO/OIE/WHO) commitment and support. The development of highly sensitive and specific point-of-care tests for use in humans and pigs remain crucial if we are to move from control towards elimination of this Neglected Tropical Disease.

*by ‘tool-kit of interventions’ we refer to standardised measures which could be utilised individually or in combination as part of an overall control programme.

## Abbreviations

FAO: Food and Agriculture Organization
NCC: Neurocysticercosis
OIE: World Organisation for Animal Health
SMART: Specific, Measurable, Achievable/attainable, Relevant, Time-bound
WHO: World Health Organization
